# The Effects of a Ketogenic Diet on Exercise Metabolism and Physical Performance in Off-Road Cyclists

**DOI:** 10.3390/nu6072493

**Published:** 2014-06-27

**Authors:** Adam Zajac, Stanisław Poprzecki, Adam Maszczyk, Miłosz Czuba, Małgorzata Michalczyk, Grzegorz Zydek

**Affiliations:** 1 Department of Sports Training—Academy of Physical Education im. J. Kukuczki in Katowice, Mikolowska 72a, 40-065 Katowice, Poland; E-Mails: a.zajac@awf.katowice.pl (A.Z.); m.czuba@awf.katowice.pl (M.C.); 2 Department of Biochemistry—Academy of Physical Education im. J. Kukuczki in Katowice, Mikolowska 72a, 40-065 Katowice, Poland; E-Mail: s.poprzecki@awf.katowice.pl; 3 Department of Sports Nutrition—Academy of Physical Education im. J. Kukuczki in Katowice, Mikolowska 72a, 40-065 Katowice, Poland; E-Mails: m.michalczyk@awf.katowice.pl (M.M.); g.zydek@awf.katowice.pl (G.Z.)

**Keywords:** free fatty acids, diet, aerobic capacity, insulin, cortisol, lactate concentration

## Abstract

The main objective of this research was to determine the effects of a long-term ketogenic diet, rich in polyunsaturated fatty acids, on aerobic performance and exercise metabolism in off-road cyclists. Additionally, the effects of this diet on body mass and body composition were evaluated, as well as those that occurred in the lipid and lipoprotein profiles due to the dietary intervention. The research material included eight male subjects, aged 28.3 ± 3.9 years, with at least five years of training experience that competed in off-road cycling. Each cyclist performed a continuous exercise protocol on a cycloergometer with varied intensity, after a mixed and ketogenic diet in a crossover design. The ketogenic diet stimulated favorable changes in body mass and body composition, as well as in the lipid and lipoprotein profiles. Important findings of the present study include a significant increase in the relative values of maximal oxygen uptake (VO_2max_) and oxygen uptake at lactate threshold (VO_2_ LT) after the ketogenic diet, which can be explained by reductions in body mass and fat mass and/or the greater oxygen uptake necessary to obtain the same energy yield as on a mixed diet, due to increased fat oxidation or by enhanced sympathetic activation. The max work load and the work load at lactate threshold were significantly higher after the mixed diet. The values of the respiratory exchange ratio (RER) were significantly lower at rest and during particular stages of the exercise protocol following the ketogenic diet. The heart rate (HR) and oxygen uptake were significantly higher at rest and during the first three stages of exercise after the ketogenic diet, while the reverse was true during the last stage of the exercise protocol conducted with maximal intensity. Creatine kinase (CK) and lactate dehydrogenase (LDH) activity were significantly lower at rest and during particular stages of the 105-min exercise protocol following the low carbohydrate ketogenic diet. The alterations in insulin and cortisol concentrations due to the dietary intervention confirm the concept that the glucostatic mechanism controls the hormonal and metabolic responses to exercise.

## 1. Introduction

An interaction between exercise-induced responses and nutrient availability has long been recognized [1]. It seems that altering the substrate supply during exercise can modify a training impulse, yet it has not been clearly determined to what extent. Skeletal muscle energy status exerts profound effects on resting metabolism and fuel use during exercise, exercise capacity, regulation of cell signaling and gene expression, as well as numerous processes involved in training adaptation. Some of the more recent studies on nutrition and exercise metabolism have attempted to examine scientific evidence for the hypothesis that endurance training undertaken with low carbohydrate availability promotes greater adaptive changes compared to high carbohydrate availability [1,2,3].

Athletes in endurance sports, lasting one hour or more, are constantly searching for new nutrition strategies to enhance performance. Knowledge on energy metabolism has placed the focus on dietary carbohydrates in the past 3–4 decades, with most athletes experiencing carbohydrate loading for different periods of time before competition [4,5,6]. High carbohydrate diets increase muscle and liver glycogen stores, improving endurance performance, yet at the same time, they increase the rate of carbohydrate utilization during exercise. Having this in mind, scientists and athletes have begun experimenting with dietary procedures that would decrease the rate of carbohydrate utilization, while increasing fat metabolism during prolonged physical work [7,8,9]. It seems that such an alternative in exercise metabolism can be induced by a high fat, low carbohydrate diet. Very low carbohydrate ketogenic diets have been used for years in fighting obesity and different common and rare disease states [10].

Research suggests that mild ketosis may offer therapeutic potential in diseases related to substrate insufficiency or insulin resistance, those resulting from free radical damage and hypoxia [10,11]. On the other hand, there are some data indicating that blood ketones are related to fatigue and perceived effort during exercise [12]. Most of these studies have been conducted with untrained and/or obese subjects. Research with competitive athletes in different sport disciplines is scarce, and conflicting results have been presented. Several studies with competitive athletes have indicated that low carbohydrate ketogenic diets do not compromise aerobic endurance and explosive strength performance, while decreasing body weight and fat mass [11,13,14]. Most studies with endurance athletes have indicated that prolonged ketosis results in an adaptation, after which free fatty acids become the major metabolic fuel, and carbohydrate utilization is markedly reduced during moderate, but exhausting exercise [7,9,15,16]. Thus, the justification for a low carbohydrate, high fat diet in endurance sports is to utilize a more concentrated fuel source to slow down the rate of carbohydrate use during exercise [15,16].

Having this in mind, new concepts of improving endurance performance have been created, with the hypothesis that a low carbohydrate high fat diet will increase the rate of free fatty acid (FFA) metabolism during exercise, while muscle glycogen will be preserved for later stages of an event, especially for the more intense parts [9,13,17]. The major drawback in fat loading is the fact that per unit of time, more ATP can be generated from carbohydrate than from fat oxidation. When blood-borne FFA are oxidized, the maximum rate of ATP resynthesis is about 0.40 moL/min, while an aerobic or anaerobic breakdown of glycogen can generate from 1.0 to 2.0 mol of ATP/min [18,19]. During high intensity exercise, the rate of ATP breakdown is too high to be matched by the rate of ATP synthesis from FFA. This phenomenon limits the use of fat loading in sport disciplines that require high intensity efforts from the athletes. High intensity exercise also suppresses lipolysis, thereby reducing the availability of fatty acids to the muscles [20]. An increased rate of glycolysis and lactate production during exercise also hinder the oxidation of fat by reducing the entry of long chain fatty acids into the mitochondria [21].

A major metabolic adaptation to endurance training is an increased capacity for fat oxidation [15]. Cross country cycling is a predominantly endurance sport event in which training sessions last from 1 to 4 h. The intensity of effort in this sport discipline varies from low to maximal; thus, both metabolic pathways improve significantly with training. The contribution of fat to the total energy expenditure increases after endurance training at both relative and absolute exercise intensity [22]. Most importantly, the trained muscles of athletes have a greater mitochondrial and capillary density, which enables them to oxidize more fat compared to the untrained muscles of sedentary subjects [17]. This sparing of glycogen effect allows endurance athletes to exercise longer before experiencing glycogen depletion and associated fatigue. Another important adaptive mechanism to endurance training includes increased activity of hormone-sensitive lipase (HSL) and decreased secretion of insulin, both at rest and during exercise [9,17].

Endurance trained individuals deliver more blood and oxygen to the working muscles due to a higher cardiac output and an increased arteriovenous oxygen difference. These athletes also produce less lactate at the same load due to a higher lactate threshold. Both of these adaptive changes facilitate fat oxidation. Theoretically, since endurance athletes can metabolize fat more efficiently, low carbohydrate, high fat diets should be preferred to carbohydrate loading as a nutritional strategy for increased performance. Numerous studies examining the benefits of these dietary procedures with athletes of different sport disciplines and different sports level have given conflicting results [4,13,14,16,22,23,24]. A ketogenic diet is high in fat and low in carbohydrate and protein content. Although the “ketogenic diet” is deficient in one nutrient (carbohydrates), it provides an alternative fuel source for the brain and skeletal muscles, which includes ketones. These ketone bodies include β-hydroxybutyrate and acetoacetate. A ketogenic diet may be recommended in neurological diseases (epileptogenesis), during the disruption of GLUT-1 transporters and during the low activity of pyruvate dehydrogenase (E1).

Despite some therapeutic benefits, ketogenic diets create several physiological consequences of which the most significant for physical exercise includes ketosis. Other side effects of ketogenic diets for sport performance include dehydration, hypoglycemia and increased risk of kidney stones [25,26,27]. Additionally, high fat, low carbohydrate ketogenic diets may induce metabolic disturbances, causing acidosis, weight loss, inadequate growth, hyperlipidemia, vitamin and trace elements deficiency (zinc, selenium and copper), hypoglycemia, hyperuricemia, anemia and leukopenia [27].

### Objective of the Research and Main Hypothesis

The main objective of this research was to determine the effects of a long-term, low carbohydrate, ketogenic diet, rich in polyunsaturated fatty acids, on aerobic performance in off-road cyclists. Additionally, the effects of this diet on body mass and body composition were evaluated, as well as those that occurred in the lipid and lipoprotein profiles, due to the dietary intervention. The effects of the high fat diet on resting and exercise concentrations of chosen hormones and metabolites were also determined. The main hypothesis stated that a long-term, low carbohydrate, ketogenic diet, applied in off-road cyclists, would decrease body mass and body fat content, while increasing FFA metabolism during continuous exercise with moderate intensity, with a concomitant decrease in insulin levels and glucose uptake. It was also hypothesized that the ketogenic diet would allow the subjects to maintain the level of aerobic power and capacity as evaluated by the value of maximal oxygen uptake (VO_2max_) and lactate threshold (LT). The study was approved by the Bioethical Committee for Research at the Academy of Physical Education in Katowice.

## 2. Material and Methods

### 2.1. Subjects

The research material included 8 male subjects, aged 28.3 ± 3.9 years, who competed in off-road cycling with a training experience of at least 5 years and a minimal VO_2max_ of 55 mL/kg/min. All of the study participants were informed of the objective of the experiment and the accompanying risks. Table 1 presents the baseline somatic and morphological characteristics of the off-road cyclists participating in the experiment.

### 2.2. Experimental Design

The experiment consisted of two distinct phases and was conducted during the preparatory period of the annual training cycle, where a high volume of work dominated the daily training loads. Each testing phase lasted 3 days and was preceded by either 4 weeks of a mixed diet or a low carbohydrate-ketogenic diet. We adopted a crossover design, with the athletes randomly assigned to either the mixed or ketogenic diets for the first month, with a change of feeding procedures during the second month, while similar training loads were adopted by all cyclists during the study period. The training protocol included work of high volume and moderate intensity. There was a 1 week recovery macrocycle incorporated between the two monthly dietary and training interventions without feeding restrictions. On the second day of the evaluations, a progressive cycloergometer test was administered to determine maximal oxygen uptake and the level of lactate threshold (LT). The progressive test was performed on an Excalibur Sport cycloergometer (Lode BV, Groningen, The Netherlands), beginning with a workload of 80 W, which was increased by 40 W every 3 min until volitional exhaustion. If a subject terminated the test before completing the given workload, then the maximum workload was calculated from the formula WR_max_ = WR_k_ + (*t*/*T* × WR_p_) [28], where WR_k_ is the previous workload, *t* the exercise duration with the workload until premature failure, *T* the duration of each workload and WR_p_ the amount of workload by which exercise intensity increased during the test. During the progressive test, the following variables were constantly registered: heart rate (HR, bts/min), oxygen uptake (VO_2_, mL/kg/min) and expired carbon dioxide (CO_2_) (MetaLyzer 3B-R2, Cortex, Berlin, Germany). Fingertip capillary blood samples for the assessment of lactate (LA, mmol/L) concentration (Biosen C-line Clinic, EKF-diagnostic GmbH, Barleben, Germany) were drawn at rest and at the end of each step of the test, as well as during the 3, 6, 9 and 12 min of recovery. The lactate threshold was determined by the D-max method [29]. Our earlier study [30] demonstrated that LT determined by the D-max method corresponds to the maximal lactate steady state (MLSS).

**Table 1:** The somatic and morphological characteristics of off-road cyclists taking part in the experiment.

Variables	X	SD
Body mass (kg)	80.34	7.36
Body height(cm)	179.78	8.06
BMI (kg/m^2^)	24.93	3.01
Fat mass (kg)	11.71	5.57
WBC (10^3^/μL)	6.15	1.77
RBC (10^6^/μL)	5.31	0.29
Hematocrit (%)	45.17	3.43
Hemoglobin (g/dL)	15.23	0.93

On the third day of each testing session, each cyclist performed a continuous effort on a cycloergometer with varied intensity, with a duration of 105 min. During the first 90 min of the test protocol, the intensity was set at 85% of the earlier determined LT expressed in watts. The last 15 min of the continuous effort were conducted at maximum individual intensity with the load set to 115% of LT. During the first phase of research, the test protocol was performed 3 h after a mixed meal, while in the second phase, following a high fat meal, both at an equal energetic value of 600 kcal. Venous blood samples were taken at rest, after 45 and 90 min of exercise at moderate intensity and immediately after the cessation of the maximal effort in the 105 min of the exercise protocol.

Body mass and composition were evaluated in the morning hours (7–8 am) after an overnight fast, with the electrical impedance method (Inbody 720, Biospace Co., Tokyo, Japan) before each phase of the experiment. Food and liquid intake was monitored the night before these measurements.

### 2.3. Diet Composition

During initial testing, a dietary interview (3-day recall: 2 workdays and 1 weekend day) was carried out in order to determine the composition and caloric value of the diet used by the off-road cyclists. Afterwards, isocaloric diets were composed for both phases of the experiment by a certified dietician with the use of a computer program, Diet 5, recommended by the Polish Institute of Nutrition. The mixed or standard Western diet included 50% carbohydrates, 30% fats and 20% protein, while the ketogenic diet was composed of 70% fat, 15% protein and 15% carbohydrates. The composition of the diets used in the experiment is presented in Table 2, specifying the proportions of basic nutrients, and the amount of particular types of fats in each diet. The proportions of saturated and unsaturated fatty acids and especially the amount of omega-3 and omega-6 fatty acids were reported in detail, because of the potential effect on vasodilation, muscle damage and muscle inflammation, erythrocyte deformability, blood viscosity and oxidative stress [31,32]. The average caloric value of the diets equaled 3865 ± 156 kcal.

**Table 2:** Diet composition of the off-road cyclists with particular types of fatty acids. Ket, ketogenic.

Diet	Mix	Ket
Carbohydrate (CHO)	50%	15%
Fat	30%	70%
Protein (Pro)	20%	15%
Saturated fatty acids (SFA)	30 g	68 g
Monounsaturated fatty acids (MUFA)	33 g	130 g
Polyunsaturated fatty acids (PUFA)	28 g	35 g
Omega-3	3.2 g	7.1 g
Omega-6	10.7 g	25.4 g

#### Biochemical Analysis

The biochemical evaluations included plasma: insulin (Ins, mlU/L), cortisol (Cor, nmoL/L), testosterone (T, ng/mL), total cholesterol (T-Ch, mg/dL) (CH 201), triglycerides (TG, mg/dL) (TR 210), high density lipoproteins (HDL-Ch, mg/dL) (CH 203) and low density lipoproteins (LDL-Ch, mg/dL) (Friedewald equation). Additionally, the resting and exercise concentrations of glucose (Glu, mg/dL) (GL 2623), free fatty acids (FFA, mmoL/L) (FA 115) and β-hydroxybutyrate (β-HGB, mmoL/L), with the participation of NAD and β-hydroxybutyrate dehydrogenase, were evaluated, as well as capillary lactate concentration (LA, mmoL/L) (LC 2389). Hormone concentrations were evaluated with the radioimmune method using commercial kits for assays and the reader: Gamma Wallec LKB (DSL 2100 IRMA, Diagnostic kits (Diagnostic System Laboratories and, Webster, TX, USA). The evaluation of the lipid and lipoprotein profiles, as well as glucose and FFA concentrations was performed with the use of Randox kits (Randox Laboratories Limited, Crumlin, United Kingdom) and Spectrophotometer Shimadzu 1200 UV-Vis (Shimadzu Schweiz GmbH., Reinach BL, Switzerland). All biochemical analyses were performed in duplicate measurements, with the mean intra-assay variability ranging from 0.86 to 0.95.

### 2.4. Statistical Analysis

The descriptive analyses consisted of the mean and standard deviation. For all measured variables, the estimated sphericity was verified according to the Mauchly’s W test, and the Greenhouse–Geisser correction was used when necessary. Before using parametric tests, the assumption of normality was verified using the Kolmogorov–Smirnov test. The comparison of analyzed values before and after the introduction of the experimental factor, was carried out with a two-way repeated measures ANOVA. When significant differences were found, the Tukey HSD *post hoc* tests were used. The effect size (eta-squared; η^2^) of each test was calculated for all analyses. The effect size was classified according to Hopkins [33]. Statistical significance was set at *p* < 0.05. The data were analyzed using Statistica 9.1 software (StatSoft Sp. z o o., Krakow, Poland).

## 3. Results

A two-way repeated measures ANOVA revealed a statistically significant effect of the diet intervention program on β-hydroxybutyrate concentration evaluated at rest (β-HGB). Tukey’s HSD *post hoc* test revealed a statistically significant increase, from 0.04 mmoL/L (mixed), to 0.15 mmoL/L, after the ketogenic diet in β-HGB concentration (*F* = 18.45, η^2^ = 0.615, *p* = 0.001).

Table 3 presents the characteristics of body mass and body composition after the mixed and ketogenic diets, while, Table 4 shows the lipid and lipoprotein profiles during the mixed and ketogenic diets at rest and during the exercise protocol.

**Table 3:** Body mass and body composition in off-road cyclists after a mixed (Mix) and ketogenic (Ket) diet.

Variables	Mix		Ket		η^2^	*p*
	X	SD	X	SD		
Body mass (kg)	80.14	7.26	78.26	7.86	0.552	0.011
BMI (kg/m^2^)	24.87	3.09	23.89	3.10	0.471	0.012
FAT (%)	14.88	3.78	11.02	3.66	0.747	0.001

The diet intervention significantly differentiated T-Ch (*F* = 13.26; *p* = 0.0083), HDL-Ch (*F* = 8.12; *p* = 0.024) and LDL-Ch (*F* = 20.22; *p* = 0.0027). The exercise protocol caused statistically significant changes in the concentration of TG (*F* = 5.05; *p* = 0.0086) (HDL-Ch (*F* = 16.47; *p* = 0.0001)). A significant interaction in the concentration of TG was registered (diet × exercise) (*F* = 3.28; *p* = 0.041). Significant differences due to the diet intervention occurred during the 90 min of the exercise protocol in regard to T-Ch, (*p* < 0.01) and HDL-Ch, (*p* = 0.05), while during maximal effort, such differences occurred in LDL-Ch (*p* < 0.01) (Table 4).

Table 5 presents HR, RER and VO_2_ values after the mixed and ketogenic diets during the exercise protocol (rest, 10 min, 45 min, 90 min and max effort). A two-way repeated measures ANOVA and *post hoc* tests revealed a statistically significant effect at rest on the respiratory exchange ratio (*F* = 18.22, η^2^ = 0.601, *p* = 0.001), during the 45 min of exercise (*F* = 10.16, η^2^ = 0.442, *p* = 0.001) (90 min (*F* = 10.05, η^2^ = 0.435, *p* = 0.001)), after the ketogenic diet. No statistically significant differences were observed during maximal effort (*F* = 1.22, η^2^ = 0.101, *p* = 0.065).

**Figure 1:**
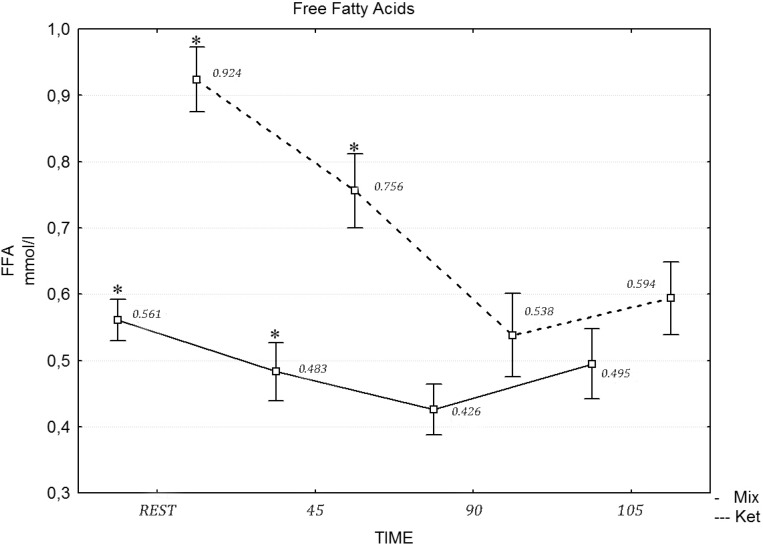
The concentration of free fatty acids (FFA) during the exercise protocol, after a mixed and ketogenic diet. * Statistical significance with *p* < 0.05.

**Figure 2:**
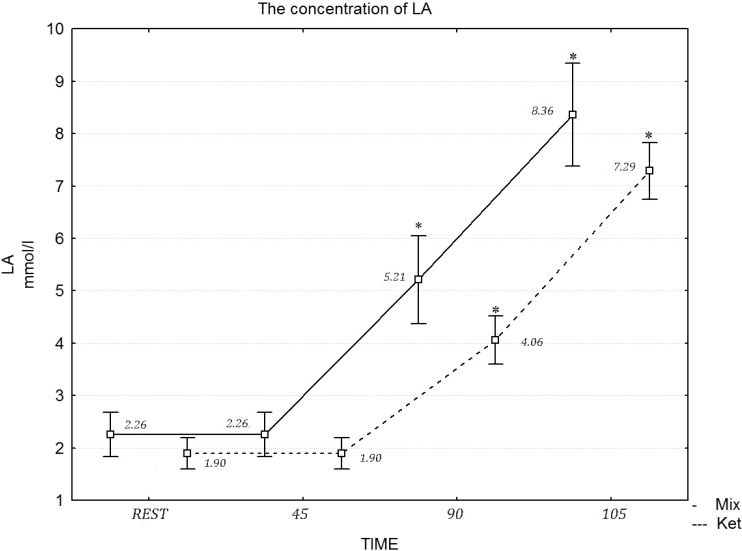
The concentration of LA during the exercise protocol, after a mixed and ketogenic diet. * Statistical significance with *p* < 0.05.

Table 6 presents the values of the biochemical variables under analysis. A two-way repeated measures ANOVA revealed a statistically significant effect of exercise on insulin (*F* = 10.70, η^2^ = 0.452, *p* = 0.015), glucose (*F* = 17.43, η^2^ = 0.591, *p* = 0.001) and cortisol (*F* = 17.21, η^2^ = 0.587, *p* = 0.001), while the differences in the FFA concentration after the mixed and ketogenic diets were statistically significant. The *post hoc* tests revealed that the concentration of FFA differed at rest (*F* = 12.13, η^2^ = 0.491, *p* = 0.021), during the 45 min of exercises (*F* = 12.22, η^2^ = 0.501, *p* = 0.012), the 90th min (*F* = 19.21, η^2^ = 0.658, *p* = 0.001) and after the maximum effort (*F* = 22.23, η^2^ = 0.747, *p* = 0.001), following the ketogenic diet (Figure 1). A two-way repeated measures ANOVA revealed no statistically significant effect of diet intervention on the testosterone (*F* = 1.14, η^2^ = 0.087, *p* = 0.061) concentration at rest and during the exercise protocol. Similarly, a two-way repeated measures ANOVA revealed a statistically significant effect of the diet in the 90 min of exercise (*F* = 21.11, η^2^ = 0.717, *p* = 0.001) and during the maximum effort in LA concentration (*F* = 20.03, η^2^ = 0.697, *p* = 0.001) (Figure 2).

A two-way repeated measures ANOVA revealed no statistically significant effect of the diet intervention on creatine kinase (CK) and lactate dehydrogenase (LDH) activity.

Table 7 presents the values of physiological variables under analysis. A two-way repeated measures ANOVA revealed statistically significant differences between the moderate and small size effects of mixed and ketogenic diets on VO_2max_ (*F* = 23.70, η^2^ = 0.751, *p* = 0.001), VO_2_LT (*F* = 17.43, η^2^ = 0.592, *p* = 0.012), LT work load (*F* = 14.21, η^2^ = 0.548, *p* = 0.015) and max work load (*F* = 10.11, η^2^ = 0. 491, *p* = 0.037). *Post hoc* tests revealed a statistically significant effect of diet on VO_2max_ (*p* = 0.001), VO_2_LT (*p* = 0.001), LT work load (*p* = 0.001) and maximal work load (*p* = 0.012).

**Table 4:** Lipid and lipoprotein profiles during the mixed and ketogenic diets at rest and during the exercise protocol.

Variables	Rest			45 min			90 min			Max Effort		
	Mix	Ket	*p*	Mix	Ket	*p*	Mix	Ket	*P*	Mix	Ket	*p*
Triglycerides (mg/dL)	117.21 ± 10.11	90.11 ± 8.75	0.002 *	108.24 ± 8.23	110.23 ± 8.34	0.041	125.11 ± 9.33	129.96 ± 9.41	0.058	129.86 ± 9.44.	112.34 ± 8.51	0.001 *
Total cholesterol (mg/dL)	188.34 ± 16.22	215.34 ± 19.54	0.001 *	196.31 ± 16.32	223.61 ± 20.12	0.001 *	190.78 ± 17.34	230.93 ± 20.15	0.001 *	191.67 ± 17.57	226.11 ± 20.45	0.001 *
High density lipoproteins (mg/dL)	96.51 ± 7.12	117.20 ± 9.14	0.002 *	99.12 ± 7.09	115.28 ± 9.10	0.002 *	92.45 ± 7.031	118.34 ± 9.45	0.001 *	91.89 ± 7.01	119.97 ± 9.51	0.001 *
Low density lipoproteins (mg/dL)	69.12 ± 4.21	74.58 ± 5.12	0.461	73.31 ± 5.11	80.78 ± 6.45	0.049	72.28 ± 5.09	86.21 ± 6.75	0.068	72.22 ± 5.02	83.43 ± 6.56	0.593

* statistical significance with *p* < 0.05.

**Table 5:** Heart rate (HR), respiratory exchange ratio (RER) and VO_2_ values after a mixed and ketogenic diet during the exercise protocol.

Variable	Rest		10 min		45 min		90 min		Max Effort	
	Mix	Ket	Mix	Ket	Mix	Ket	Mix	Ket	Mix	Ket
HR	72 ± 5	75 ± 6	150 ± 3	150 ± 3	158 ± 4	161 ± 5	167 ± 5	169 ± 5	187 ± 6	185 ± 6
VO_2_	7.20 ± 1.22	9.40 ± 1.41	35.37 ± 3.45	41.25 ± 4.22	37.50 ± 3.64	44.25 ± 4.56	40.87 ± 4.11	44.00 ± 4.41	51.00 ± 4.87	50.00 ± 4.85
RER	0.88 ± 0.04	0.76 ± 0.01	0.86 ± 0.04	0.78 ± 0.02	0.85 ± 0.04	0.79 ± 0.02	0.84 ± 0.03	0.79 ± 0.02	0.97 ± 0.05	0.94 ± 0.05

**Table 6:** Values of biochemical variables (insulin (Ins), glucose (Glu), creatine kinase and lactate dehydrogenase activity), as well as testosterone (T) and cortisol (Cor) concentration after a mixed and ketogenic diet at rest and during the exercise protocol.

Variables	Rest		45 min		90 min		Max Effort	
	Mix	Ket	Mix	Ket	Mix	Ket	Mix	Ket
Ins (U/L)	19.21 ± 0.81	9.87 ± 0.45	6.02 ± 0.31	4.25 ± 0.22	5.45 ± 0.25	4.97 ± 0.29	9.89 ± 0.45	5.63 ± 0.29
Glu (mg/dL)	91.26 ± 4.11	91.32 ± 4.13	106.11 ± 4.98	98.61 ± 4.22	89.78 ± 4.01	90.04 ± 4.07	121.67 ± 5.14	119.41 ± 5.08
CK (U/L)	126.32 ± 10.22	119.45 ± 9.74	158.12 ± 11.21	129.11 ± 10.31	160.76 ± 13.24	139.34 ± 10.58	178.12 ± 15.45	140.07 ± 12.51
LDH (U/L)	321.26 ± 30.14	262.23 ± 24.24	349.56 ± 32.17	267.56 ± 24.52	359.65 ± 33.23	265.45 ± 24.45	439.76 ± 39.56	311.21 ± 27.61
T (ng/L)	6.12 ± 0.4	5.86 ± 0.3	8.78 ± 0.6	7.21 ± 0.5	9.38 ± 0.7	8.08 ± 0.6	7.91 ± 0.5	8.14 ± 0.6
Cor (nmol/L)	649 ± 62	553 ± 49	389 ± 29	435 ± 33	495 ± 38	579 ± 51	650 ± 62	676 ± 65

**Table 7:** Physiological variables (max work load, VO_2_max, VO_2_LT and LT work load) in off-road cyclists after a mixed (Mix) and ketogenic (Ket) diet.

Variables	Mix		Ket		*p*
	X	SD	X	SD	
Max work load (W)	362	16.09	350	14.60	0.037
VO_2max_ (mL/kg/min)	56.02	3.50	59.40	3.10	0.001
VO_2_LT (mL/kg/min)	43.50	1.80	47.80	2.10	0.012
LT work load (W)	257	10.60	246	9.50	0.015

## 4. Discussion

Consuming a low carbohydrate ketogenic diet may be recommended to promote fat oxidation during exercise at moderate intensity and at rest. Fat loading may also slow down the rate of carbohydrate utilization and enhance endurance performance in long distance events lasting from 2 to 5 h. This is especially true during long-term ketogenic diets, where the body adapts to increased fat oxidation through enzymatic and endocrine changes [34,35]. The low carbohydrate, high fat diet applied in this study caused a four-fold elevation of pre-exercise β-hydroxybutyrate concentration and a two-fold increase in resting plasma FFA concentration. This indicates compliance with the prescribed ketogenic diet. Compared to a high carbohydrate diet, or a mixed diet with 50%–70% of energy coming from carbohydrate (CHO), a high fat diet with 70% of the calories derived from fat significantly increased the contribution of FFA to the total energy expenditure during moderate intensity exercise. This was observed during the first 90 min of the exercise protocol. During the last 15 min of exercise, when maximal intensity was introduced, FFA metabolism was inhibited by glycolysis, which was evidenced by significant increases in LA concentration. This phenomena was observed in the case of both diets, yet it was more pronounced in the case of the high fat diet [21,22].

The benefits of a ketogenic diet related to athletic performance may also be caused by changes in body mass and body composition. This may be of significance, not only in aerobic endurance sport disciplines, but also in sports that include weight class divisions and require body mass control and management [11]. The ketogenic diet introduced in this research project stimulated favorable changes in body mass and body composition, as well as in the lipid and lipoprotein profiles. The most likely reason for such changes included the predominance of polyunsaturated fatty acids in such a diet [34,35,36]. Further benefits of a high fat diet, with a significant intake of Ω-3 fatty acids, may be related to reduced post exercise muscle damage, which was observed by lower rest and exercise plasma CK and LDH activity in this research project [37]. These differences were especially visible after the maximal effort phase.

During long endurance exercise at moderate intensity (between 50% and 70% VO_2max_), a lower RER and lactate concentration are observed, yet a higher HR and VO_2_ following a high fat diet, compared to a mixed or high carbohydrate diet [34]. A significantly lower respiratory exchange ratio at submaximal workloads after the ketogenic diet indicates increased lipid metabolism. The ketogenic diet applied in the present study resulted in lower plasma lactate concentrations at rest, during the moderate intensity continuous exercise and especially after the last 15 min of the exercise protocol performed with maximal effort. Important findings of the present study include a significant improvement in relative values of VO_2max_ and LT VO_2_ after the ketogenic diet, which can be explained by reductions in body mass and fat mass and or greater oxygen uptake necessary to obtain the same energy yield as on a mixed diet due to increased fat oxidation or by enhanced sympathetic activation [38,39]. Previous investigations have also reported a shift in LT to higher workloads under conditions of glycogen store reductions, due to a low carbohydrate diet, fasting or exhausting exercise [17]. This phenomenon has not been fully explained, and there is still a debate on whether a ketogenic diet induced a shift in the LT and whether a reduction in the maximal LA concentration depends on a decreased rate of glycolysis or an inhibited lactate efflux from working muscles due to reduced blood buffering capacity [17]. Most research projects indicate a tendency towards lower blood pH and reduced blood base excess and bicarbonate levels after a ketogenic diet at rest; and especially after exercise with maximal intensity [34]. Our research showed improvements in VO_2max_ and VO_2_LT, yet the power output during work at maximal intensity was compromised on the ketogenic diet, which can be explained by lower muscle glycogen stores and the reduced activity of glycolytic enzymes due to the four-week diet intervention [19,40]. The increase in aerobic capacity following the ketogenic diet may also have been influenced by positive changes in the morphological characteristics of the off-road cyclists, where higher values of RBC, HCT and HGB were registered at rest and during the exercise protocol. This could be explained by the ergogenic effects of Ω-3 polyunsaturated fatty acids on erythrocyte membrane integrity, erythrocyte deformability and blood viscosity, factors that improve circulation and oxygen transport to working muscles [39,41].

The changes in the considered hormone concentrations induced by the ketogenic diet and the exercise protocol were similar to those reported previously in regard to insulin and cortisol, while lower values of testosterone after the high fat diet at rest and during the exercise protocol are difficult to explain. The alterations in insulin and cortisol concentrations due to the dietary intervention confirm the concept that the glucostatic mechanism controls the hormonal and metabolic responses to exercise. According to this concept, depletion of muscle and liver glycogen leads to the stimulation of lipolysis and glucose production, due to changes in the secretion of glucoregulatory hormones [37]. The main limitation of this study includes a small number of subjects participating in the experiment, the use of electrical impedance for body composition analysis and the lack of a 2–3-day carbohydrate loading phase following the four-week ketogenic diet. This could confirm or reject the hypothesis regarding improved endurance performance and increased lipid metabolism after a ketogenic diet followed by carbohydrate loading [41].

## 5. Conclusions

It can be concluded that long-term, high fat diets may be favorable for aerobic endurance athletes, during the preparatory season, when a high volume and low to moderate intensity of training loads predominate in the training process. High volume training on a ketogenic diet increases fat metabolism during exercise, reduces body mass and fat content and decreases post exercise muscle damage. Low carbohydrate ketogenic diets decrease the ability to perform high intensity work, due to decreased glycogen muscle stores and the lower activity of glycolytic enzymes, which is evidenced by a lower LA concentration and a maximal work load during the last 15 min of the high intensity stage of the exercise protocol [42].
